# Probiotic Supplementation in Preterm Infants Does Not Affect the Risk of Bronchopulmonary Dysplasia: A Meta-Analysis of Randomized Controlled Trials

**DOI:** 10.3390/nu9111197

**Published:** 2017-10-31

**Authors:** Eduardo Villamor-Martínez, Maria Pierro, Giacomo Cavallaro, Fabio Mosca, Boris Kramer, Eduardo Villamor

**Affiliations:** 1 Department of Pediatrics, Maastricht University Medical Center (MUMC+), School for Oncology and Developmental Biology (GROW), 6202 AZ Maastricht, The Netherlands; eduardo.villamormartinez@mumc.nl (E.V.-M.); b.kramer@mumc.nl (B.K.); 2 Neonatal Intensive Care Unit, Alessandro Manzoni Hospital, 23900 Lecco, Italy; maria.pierro93@gmail.com; 3 Neonatal Intensive Care Unit, Department of Clinical Sciences and Community Health, Fondazione IRCCS Cà Granda Ospedale Maggiore Policlinico, Università degli Studi di Milano, 20122 Milan, Italy; giacomo.cavallaro@mangiagalli.it (G.C.); fabio.mosca@mangiagalli.it (F.M.)

**Keywords:** probiotics, bronchopulmonary dysplasia, sepsis, necrotizing enterocolitis

## Abstract

Probiotic supplementation reduces the risk of necrotizing enterocolitis (NEC) and late-onset sepsis (LOS) in preterm infants, but it remains to be determined whether this reduction translates into a reduction of other complications. We conducted a systematic review and meta-analysis to evaluate the possible role of probiotics in altering the risk of bronchopulmonary dysplasia (BPD). Fifteen randomized controlled trials (4782 infants; probiotics: 2406) were included. None of the included studies assessed BPD as the primary outcome. Meta-analysis confirmed a significant reduction of NEC (risk ratio (RR) 0.52, 95% confidence interval (CI) 0.33 to 0.81, *p* = 0.004; random effects model), and an almost significant reduction of LOS (RR 0.82, 95% CI 0.65 to 1.03, *p* = 0.084). In contrast, meta-analysis could not demonstrate a significant effect of probiotics on BPD, defined either as oxygen dependency at 28 days of life (RR 1.01, 95% CI 0.91 to 1.11, *p* = 0.900, 6 studies) or at 36 weeks of postmenstrual age (RR 1.07, 95% CI 0.96 to 1.20, *p* = 0.203, 12 studies). Meta-regression did not show any significant association between the RR for NEC or LOS and the RR for BPD. In conclusion, our results suggest that NEC and LOS prevention by probiotics does not affect the risk of developing BPD in preterm infants.

## 1. Introduction

Bronchopulmonary dysplasia (BPD), a chronic lung disease of prematurity, is considered one of the major complications of premature birth [1,2,3,4]. The incidence of BPD is inversely proportional to gestational age, with rates reaching up to 60–90% in extremely preterm infants (22–25 weeks gestation). Infants suffering from BPD are at increased risk of death and long-term pulmonary and neurodevelopmental morbidities [5,6,7].

The pathogenesis of BPD is initiated by the arrest in alveolar and lung vascular development, due to premature birth, and sustained by inflammatory events that play a paramount role in the progression of BPD [3,4,8,9]. The initiation of the inflammatory response can already occur in utero, in the setting of chorioamnionitis [3,4,10,11]. Nevertheless, postnatal stimuli, such as the ex-utero higher oxygen partial pressures, the need for oxygen administration or mechanical ventilation, and the occurrence of postnatal infections (including late onset sepsis (LOS) and necrotizing enterocolitis (NEC)), perpetuate inflammation and lead to the establishment of BPD [12,13,14]. A dysregulation of the immune system, toward a sustained status of inflammation which is characteristic of very preterm infants, completes the multifactorial pathophysiological picture [15].

Several treatments, most of which focused on anti-inflammatory or homeostasis-restoring properties, have been attempted in order to prevent or treat BPD [16]. However, meta-analyses could confirm a reduction of BPD only for vitamin A and dexamethasone [16,17]. Moreover, vitamin A showed only a modest effect [17], while the use of dexamethasone is limited in preterm infants by its well-known long- and short-term side effects [18]. Adequate timing, dose, and formulation of steroid therapy is still under investigation in preterm infants at risk for BPD. Lately, regenerative medicine has received a great deal of attention as a promising therapeutic option for complications of prematurity, including BPD [19,20]. However, the knowledge of stem cell function is still incomplete, and further studies are needed to elucidate the impact of several manufacturing aspects that may determine the success or failure of this therapy [19,20]. In summary, despite the continuous advances in neonatal care, BPD remains a significant burden for the premature population, lacking a safe, effective and easily available treatment.

Probiotics are defined as live micro-organisms which, when administered in adequate amounts, confer a health benefit on the host [21,22]. Probiotic supplementation in preterm infants is one of the most studied interventions in neonatal medicine [23,24,25,26,27,28,29,30]. Many randomized controlled trials (RCTs) involving the use of probiotics have been performed in the last years. Several meta-analyses combined these RCTs and demonstrated that probiotic supplementation reduces mortality, NEC, and LOS, as well as the time to achieve full enteral feeding in preterm infants [23,24,25,26,27,28,29,30,31]. Although until now no study has been performed to analyze the effect of probiotics on BPD as primary outcome, a number of RCTs included BPD as a secondary outcome. There are several hypothetical mechanisms by which probiotics may exert a protective effect against BPD: (1) by reducing postnatal inflammatory processes such as NEC and LOS; (2) by modulating the immune function [32,33]; (3) by improving the nutritional status and growth of the infants [30,31,34]; and (4) through the antioxidant properties of probiotics [35]. Therefore, in the present systematic review we aimed to collect and analyze the current evidence on the effects of probiotic supplementation on the risk of developing BPD in preterm infants.

## 2. Materials and Methods

A protocol was developed prospectively that detailed the specific objectives, criteria for study selection, the approach to assessing study quality, clinical outcomes, and statistical methodology. The study is reported according to the PRISMA checklist [36].

### 2.1. Data Sources and Search Strategies

A comprehensive literature search was undertaken using PubMed, EMBASE and CENTRAL (the Cochrane Central Register of Controlled Trials, The Cochrane Library) from their inception to 1 July 2017. Combinations of the following terms (including MeSH terms) were used to search for relevant publications: (probiotic(s) OR lactobacillus OR saccharomyces OR bifidobacterium OR streptococcus) AND (“preterm infant” OR “premature infant” OR “extremely low birth weight infant” OR “very low birth weight infant”). Language was not restricted. Additional strategies to identify studies included manual review of reference lists of key articles that fulfilled our eligibility criteria, use of the “related articles” feature in PubMed, use of the “cited by” tool in Web of Science and Google Scholar, and manual review of reference lists of meta-analyses on probiotics in preterm infants [23,24,25,27,28,30,34,37,38,39,40,41,42,43,44]. The search method used to identify all relevant articles was discussed and developed by two authors (EV-M and EV) and the final search string was approved by all authors.

### 2.2. Eligibility Criteria and Study Selection

The initial search was performed by two reviewers (EV-M and EV), who eliminated clearly irrelevant articles based on the title and abstract as defined by the pre-set selection criteria. The final selection of articles was made by mutual consideration of both authors. Studies were included if they were RCTs involving the use of probiotics in preterm infants (gestational age, GA < 37 weeks) and reported results on BPD. BPD was defined as dependence on supplementary oxygen either at 28 days of life (BPD28) or at a postmenstrual age (PMA) of 36 weeks (BPD36) [2]. However, the use of another BPD definition was not an exclusion criterion.

Studies were reviewed to ensure that study populations did not overlap by checking subject sources and studying time-frame. Where two or more studies reported on the same population, the most recent study was preferentially used (provided it reported data on BPD) to avoid duplicate data.

### 2.3. Data Extraction and Assessment of Risk of Bias

Two groups of investigators (EV-M/EV and MP/GC) extracted the data independently by using a data collection form designed for this review. Data extracted included: gestational age (GA) and birth weight (BW) of participants, patient inclusion criteria, study design (age at the first day of intervention, duration of intervention, dosage, and type of probiotic), and outcomes of interest (BPD, LOS, NEC, and mortality).

Two reviewers (EV-M and EV) independently assessed risk of bias in each trial by using the Cochrane “Risk of Bias Assessment Tool” [45]. For each domain (allocation sequence, allocation concealment, blinding of participants and outcome assessors, incomplete outcome data, selective outcome reporting, and other potential sources of bias) the risk of bias was assessed as low, high, or unclear. Potential discrepancies during the data extraction process and assessment of risk of bias were resolved by discussion and consensus among all reviewers.

### 2.4. Statistical Analysis

Studies were combined and analyzed using comprehensive meta-analysis V3.0 software (Biostat Inc., Englewood, NJ, USA). We used a random-effects model to account for anticipated heterogeneity, resulting from the differences in methodology between studies. However, analysis using a fixed-effect model was also carried out to ensure that the model used for the meta-analysis would not affect the results. Effect size was expressed as Mantel–Haenszel risk ratio (RR) and 95% confidence interval (CI). Statistical heterogeneity was assessed with the Cochran’s Q statistic and by the *I^2^* statistic, which is derived from Q and describes the proportion of total variation that is due to heterogeneity beyond chance [45]. An *I*^2^ value of 0% indicates no observed between-study heterogeneity, and large values show increasing between-study heterogeneity. The risk of publication bias was assessed by visual inspection of the funnel plot and using an Egger test. To identify any study that may have exerted a disproportionate influence on the summary effect, we calculated the summary effect excluding studies one at a time. To explore differences between studies that might be expected to influence the effect size, we performed subgroup sensitivity analysis and univariate random-effects meta-regression (method of moments) [46,47]. A potential pitfall with meta-regression analysis is that with few trials and many possible covariates, false positive findings and data dredging can happen [47]. We chose to prespecify NEC, LOS, and mortality as covariates to analyze with meta-regression to protect against this issue. A probability value of less than 0.05 (0.10 for heterogeneity) was considered statistically significant.

## 3. Results

**Figure 1:**
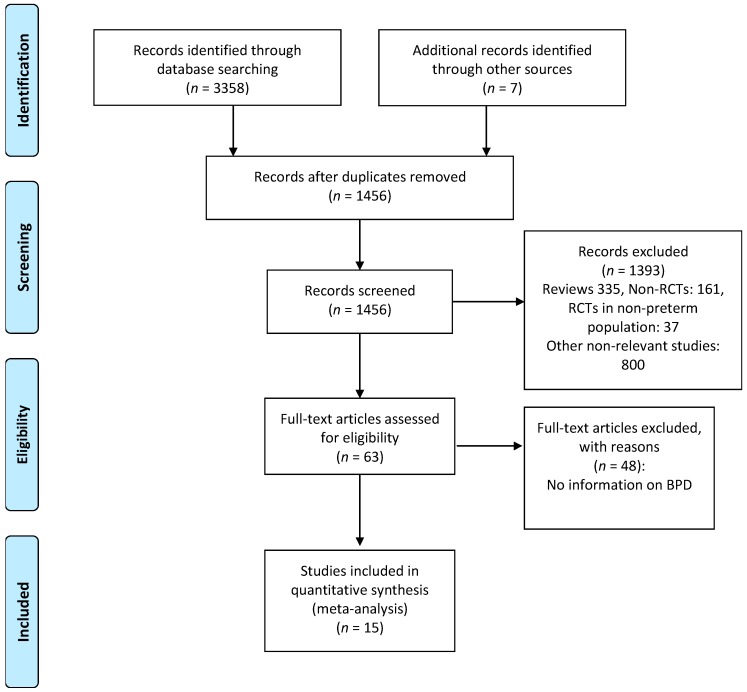
Flow diagram of literature search process. RCTs: randomized controlled trials; BPD: bronchopulmonary dysplasia.

There was no substantial disagreement between reviewers on articles for inclusion, data extraction, and risk of bias assessment. Based on the titles and abstracts of 1456 citations, we identified 63 potentially relevant studies, of which 15 met the inclusion criteria [48,49,50,51,52,53,54,55,56,57,58,59,60,61,62] (Figure 1). The main characteristics of the studies are shown in Table 1. The 15 studies included 4782 infants of which 2406 infants received probiotics. Twelve studies [48,49,50,51,53,54,55,56,57,58,60,62] included very preterm (GA < 32 weeks) and/or very low BW (VLBW) infants (<1500 g). One study [48] included extremely low BW preterm infants (<1000 g). Two studies included larger preterm infants; one [52] included infants with GA < 34 weeks and the other [59] included infants with GA < 37 weeks. The included studies randomized infants to different preparations, times of initiation, and duration of therapy (Table 1). Details of the risk of bias analysis are depicted in Appendix A, Table A1. None of the included studies reported serious adverse events potentially associated with the use of probiotics.

**Figure 2:**
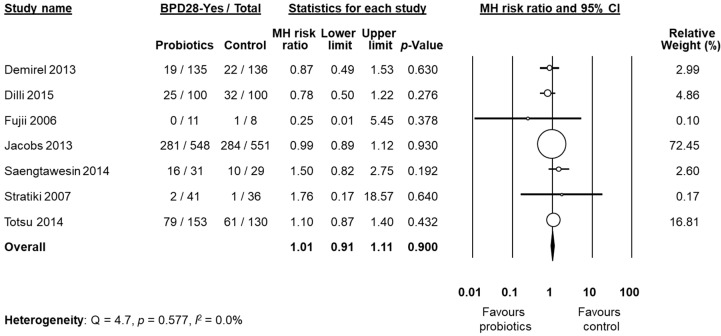
Random effects meta-analysis: Probiotic supplementation and risk of BPD28 (bronchopulmonary dysplasia, defined as oxygen dependence at 28 days of life). MH: Mantel–Haenszel; CI: confidence interval.

BPD was not the primary outcome in any of the included studies. Six studies [53,55,58,60,61,62] clearly defined BPD as BPD28 and/or BPD36, whereas nine studies did not [48,49,50,51,52,54,56,57,59]. A clarification on BPD definition was kindly provided by the authors of eight studies [48,49,50,51,52,54,56,57]. After these clarifications, data on BPD28 were available from six studies [50,51,52,53,56,60]. We decided to pool the study of Stratiki et al. [59] that did not specify a BPD definition, with studies reporting BPD28. Neither the individual studies nor the meta-analysis could detect a significant effect of probiotic supplementation on BPD28 (RR 1.01, 95% CI 0.91 to 1.11, *p* = 0.900, Figure 2). The use of a fixed effect model instead of a random effects model did not significantly affect the results of the meta-analysis (RR 1.00, 95% CI 0.91 to 1.10, *p* = 0.999). In sensitivity analyses, excluding one study at a time, the summary RR ranged from 0.99 (95% CI 0.89–1.10, *p* = 0.900), when the study of Totsu et al. [60] was excluded, to 1.04 (95% CI 0.86–1.25, *p* = 0.703), when the study of Jacobs et al. [53] was excluded (Appendix A, Table A2). The study of Fujii et al. [52] included larger infants than the other five studies (Table 1). However, when this study was excluded, overall results were not substantially affected (RR 1.01, 95% CI 0.91–1.11, *p* = 0.983). Exclusion of the study by Stratiki et al. [59], in which BPD was not clearly defined, did not significantly affect results (RR 1.01 95% CI 0.91–1.11, *p* = 0.829). Further sensitivity analysis and assessment of publication bias were not performed for BPD28 due to the low number of studies.

**Figure 3:**
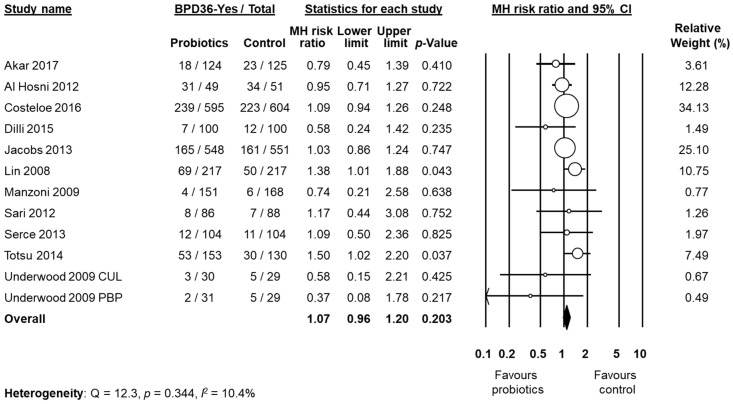
Random effects meta-analysis: Probiotic supplementation and risk of BPD36 (bronchopulmonary dysplasia, defined as oxygen dependence at 36 weeks post-menstrual age). MH: Mantel–Haenszel; CI: confidence interval. CUL: Culturelle preparation; PBP: ProBioPlus DDS preparation.

**Figure 4:**
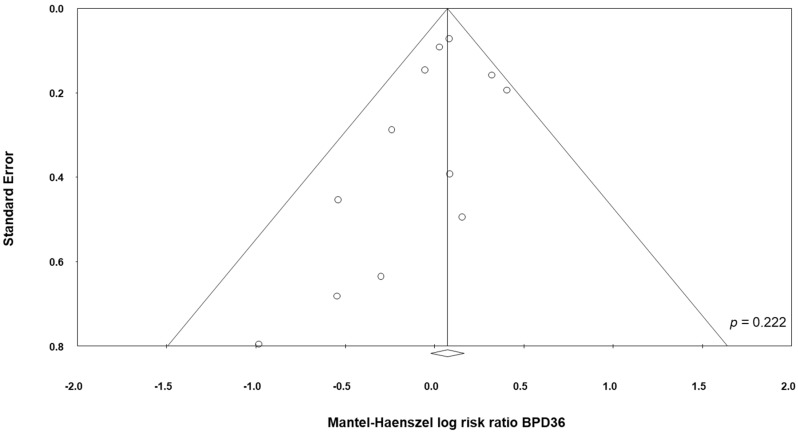
Funnel plot assessing publication bias for BPD36 (bronchopulmonary dysplasia, defined as oxygen dependence at 36 weeks post-menstrual age).

Data on BPD36 were available from 11 studies [48,49,51,53,54,55,57,58,60,61,62]. The study of Underwood et al. [61] randomized infants into three different groups: a placebo group and two treatment groups based on different probiotic preparations (Table 1). For the purposes of this analysis, the two treatment groups of the trial of Underwood et al. [61] were considered as two separate studies. The study of Lin et al. [54], showed a significant increase of the BPD36 risk in the infants receiving probiotics (RR 1.38, 95% CI 1.01 to 1.88, *p* = 0.043). In contrast, neither the other individual studies nor the meta-analysis could detect a significant effect of probiotic supplementation on BPD36 (RR 1.07, 95% CI 0.96 to 1.20, *p* = 0.203, Figure 3). Although some degree of asymmetry was observed by visual inspection of the funnel plot, Egger's test could not show any evidence of publication bias (Figure 4). The use of a fixed effect model instead of a random effects model did not significantly affect the results of the meta-analysis (RR 1.08, 95% CI 0.98 to 1.18, *p* = 0.123). In sensitivity analyses, excluding one study at a time, the summary RR ranged from 1.04 (95% CI 0.93–1.17, *p* = 0.488), when the study of Lin et al. [54] was excluded, to 1.09 (95% CI 0.97–1.23, *p* = 0.138), when the study of Al Hosni et al. [48] was excluded (Appendix A Table A3).

One study [62] also included, besides BPD36, the category severe BPD (defined as any baby at 36 weeks PMA still receiving mechanical ventilator support or in at least 30% oxygen or more than 0.1 L/min of low flow oxygen) (Table 2). They report that the probiotics group did not have a significantly different risk of severe BPD compared to the control group (RR 1.21, 95% CI 0.90 to 1.62, *p* = 0.200).

For the outcome BPD36, we conducted additional sensitivity analysis by excluding studies that had uncertain/high risk of bias in the different domains. In addition, we carried out subgroup analyses of studies where *Bifidobacterium* was part of the supplementation, studies where *Lactobacillus* was part of the supplementation, studies where multiple-strain supplements were used, studies where single-strain supplements were used, and studies where infants had a mean BW < 1250 g. No subgroup analysis could demonstrate a significant effect of probiotics on BPD36 (Table 2).

**Figure 5:**
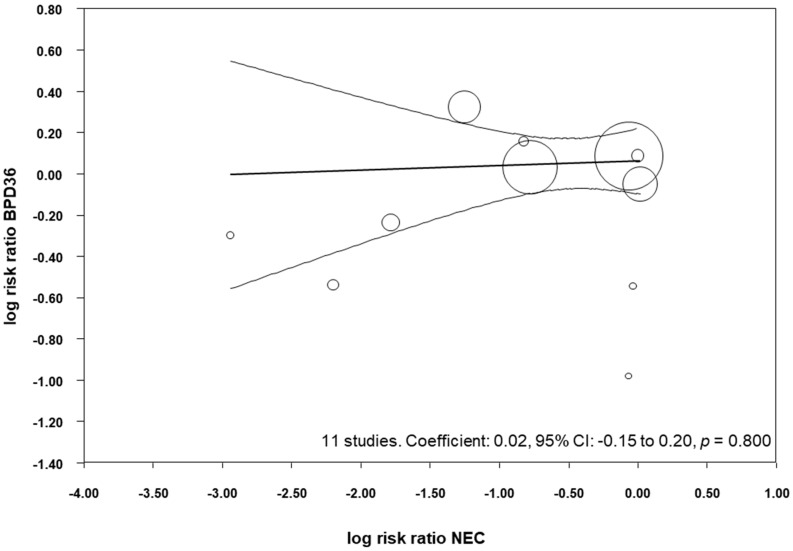
Meta-regression plot of probiotics and risk of BPD36 (bronchopulmonary dysplasia, defined as oxygen dependence at 36 weeks post-menstrual age) and probiotics and risk of necrotizing enterocolitis (NEC), CI: confidence interval.

All the included studies reported data on NEC (Table 3) and, when pooled, we observed that probiotics significantly reduced the risk of developing NEC (RR 0.52, 95% CI 0.33–0.81, *p* = 0.004, Table 4). This significant reduction of NEC was also observed when we pooled the studies that reported BPD28 (RR 0.40, 95% CI 0.18–0.88, *p* = 0.022), and when we pooled the studies that reported BPD36 (RR 0.48, 95% CI 0.29–0.81, *p* = 0.006, Table 4). We performed meta-regression analyses (methods of moments) to investigate the possible correlation between the effect size for NEC and the effect size for BPD. As shown in Figure 5, meta-regression could not detect a statistically significant correlation between the reduction in NEC produced by the probiotics and the effect size for BPD36.

**Figure 6:**
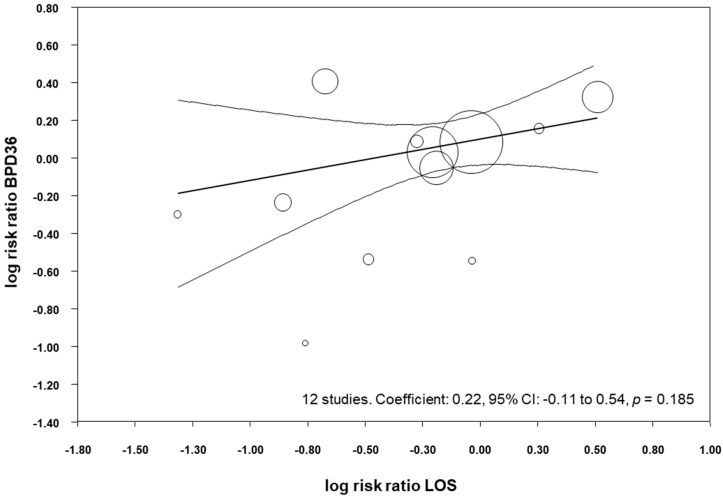
Meta-regression plot of probiotics and risk of BPD36 (bronchopulmonary dysplasia, defined as oxygen dependence at 36 weeks post-menstrual age) and probiotics and risk of late-onset sepsis (LOS), CI: confidence interval.

All the included studies reported data on LOS (Table 3), and meta-analysis demonstrated a close to significant reduction of LOS in the probiotics group (RR 0.82, 95% CI 0.65–1.03, *p* = 0.084, Table 4). Similarly, the meta-analysis of studies that reported BPD28 found a close to significant effect of probiotics on LOS (RR 0.79, 95% CI 0.63–1.00, *p* = 0.054), and the meta-analysis of studies that reported BPD36 found a close to significant reduction in LOS (RR 0.80, 95% CI 0.62–1.04, *p* = 0.090, Table 4). We performed meta-regression analyses (methods of moments) to investigate the possible correlation between the effect size for LOS and the effect size for BPD36. As shown in Figure 6, meta-regression could not detect a statistically significant correlation between the reduction in LOS produced by the probiotics and the effect size for BPD36.

All the included studies reported data on mortality (Table 3), but meta-analysis could not demonstrate a significant reduction of mortality in the probiotics group (RR 0.80, 95% CI 0.60–1.06, *p* = 0.114, Table 4). Moreover, the meta-analysis of studies that reported BPD28 could not find a significant effect of probiotics on mortality (RR 0.78, 95% CI 0.37 to 1.66, *p* = 0.518), and neither could the meta-analysis of studies that reported BPD36 (RR 0.77, 95% CI 0.56 to 1.05, *p* = 0.101). We performed meta-regression analyses (methods of moments) to investigate the possible correlation between the effect size for mortality and the effect size for BPD36. This meta-regression could not detect a statistically significant correlation between the changes in mortality produced by the probiotics and the effect size for BPD36 (coefficient 0.04, 95% CI −0.13 to 0.21, *p* = 0.638).

## 4. Discussion

Inflammatory events, such as NEC and LOS, are not only life-threatening for (very) preterm infants but also may mediate major short- and long-term adverse outcomes [63,64]. Current evidence indicates that probiotic supplementation significantly reduces NEC and LOS in preterm infants, but our data suggest that this decrease is not accompanied by a concomitant reduction in BPD. The present meta-analysis could not demonstrate any significant effect of probiotic supplementation on the risk of developing of BPD. Similarly, in a recent meta-analysis we found that probiotics did not significantly affect the risk of retinopathy of prematurity (ROP) [44]. However, our results should be interpreted with caution since the included RCTs showed relevant methodological differences in terms of enrolment criteria, timing, dose, and formulation of the probiotics used. Moreover, BPD was not the primary outcome in any of the studies and the number of RCTs of probiotics reporting on BPD as secondary outcome was relatively small. In addition, none of the included studies specifically targeted the most vulnerable population for BPD (infants < 28 weeks GA).

Inflammatory processes such as NEC and LOS may increase the risk of developing BPD through direct and indirect mechanisms. Proinflammatory cytokines may exert a direct effect on lung development or sensitize the lung to the effects of oxygen, mechanical ventilation, or other stressors [8,9,15,65]. On the other hand, infants suffering from NEC and LOS often require more aggressive and prolonged mechanical ventilation, that may lead to increased lung injury [8,9,15,65]. It has been suggested that avoiding postnatal infection is more important than avoiding invasive mechanical ventilation to decrease the inflammatory response in developing lungs [65]. Studies directed at evaluating the impact of quality improvement efforts to reduce LOS in preterm infants showed that a reduction in LOS is accompanied by decreased rates of BPD [66,67]. However, BPD is a multifactorial condition in which genetic predisposition, as well as prenatal and prenatal conditions all play a role [1,2,3,4]. In an interesting study, Lapcharoensap et al. showed a positive relationship between the reduction in LOS and the reduction in BPD with a coefficient of determination (*r*^2^) of 0.08, suggesting that only the 8% of the reduction of BPD is attributable to the reduction in nosocomial infection rates [67].

The 15 studies included in our meta-analysis represent a subset of the larger number of RCTs included in the meta-analyses on probiotics for NEC and LOS prevention. Therefore, we analysed whether the protective effects of probiotics on NEC and/or LOS were also present in the RCTs included in our study. Pooling the 15 studies showed a significant reduction of NEC (RR 0.52, 95% CI 0.33 to 0.81) and a close to significant reduction of LOS (RR 0.82 95% CI 0.65 to 1.03) in the probiotics group. We speculated that studies with higher protective effects against NEC and/or LOS would show more effect on the development of BPD. However, meta-regression did not show a significant correlation between the RR for NEC and LOS and the RR for BPD. This suggests that the reduction in postnatal inflammatory events did not translate into a reduction of BPD.

Several meta-analyses showed that probiotics reduce mortality among VLBW infants [23,25,38]. It has been suggested that improved survival of VLBW infants may result in increased numbers of patients with BPD [68]. In the group of studies included in our meta-analysis, we could not observe a significant effect of probiotics on mortality (RR 0.80, 95% CI 0.60 to 1.06). In addition, meta-regression could not show a significant correlation between the RR for mortality and the RR for BPD. Therefore, our data suggest that the effect of probiotics on mortality did not affect the rate of BPD in the RCTs. Nevertheless, a robust conclusion from meta-regression would require a larger number of included studies [46,47].

One important limitation inherent to any meta-analysis on BPD is the heterogeneity of the definition of the condition [16,69,70]. In a systematic review which included 47 RCTs of drugs for BPD, 34% did not identify the definition of BPD that was used. Of the trials that defined BPD, 22 used oxygen dependency at 36 weeks PMA, with two trials refining that definition with a test of oxygen need [16]. Fourteen trials provided data on oxygen requirement and four trials used both oxygen supplementation at 28 days and oxygen supplementation at 36 weeks PMA [16]. Similarly, in our meta-analysis only six out of 15 RCTs reported a definition of BPD. Upon request, the authors of eight studies kindly clarified their definition. Even after clarification, there was marked heterogeneity in BPD definition. As pointed out by Jobe and Bancalari [69], current definitions of BPD lack precision and do not have good predictive values for later pulmonary and neurodevelopmental outcomes. There are substantial efforts being made to develop better diagnostic criteria for BPD [69], but it will take time before these improved definitions of BPD are reflected in RCTs and meta-analyses.

As mentioned above, the RCTs included in our analysis had important differences in the type, amount, and timing of probiotic supplementation. The choice of probiotic strain(s) is crucial and meta-analyses on probiotics have been criticized because, in most of them, probiotics administered for treatment/prevention of a specific disease or condition were all evaluated together [26,71,72,73]. It is now generally accepted that different bacterial strains of the same genus and species, verified also by genomic information, may exert completely different effects on the host [72]. Separate meta-analyses analysing the effects of well-defined individual, single-strain or multiple-strain probiotic preparations appear to be more appropriate, but the important heterogeneity of the RCTs makes this approach very difficult [26,71,72,73]. We attempted to explore whether the studies using *Lactobacillus* or *Bifidobacterium* species showed a different effect on BPD. We also performed a separate analysis for multi-strain probiotics because recent meta-analyses suggest that the use of more than one strain has a stronger effect in the prevention of NEC [74]. None of these subgroup analyses suggested a significant preventive effect of probiotics on BPD. However, the number of studies included in the subgroup analysis was low, making the results inconclusive.

Besides their effect on NEC and LOS prevention, there are some other mechanisms of action ascribed to probiotics which may directly counteract the disruption of lung development prompting to BPD [26,75]. In the first place, the immature immune system of premature infants is unable to balance pro-inflammatory responses, leading to a sustained status of inflammation that contributes significantly to several neonatal diseases, including BPD [15]. A decreased number of T regulatory cells (Tregs), which constitute the anti-inflammatory lymphocytic subset, and higher proportions of activated pro-inflammatory T cells have been related with the development of BPD [76,77]. Probiotics seem to have a role in improving Treg generation, expansion and activity, while decreasing activation/proliferation of the pro-inflammatory lymphocytic subsets. These effects may result in the recovery of the immune homeostasis with polarization of the immune system toward an anti-inflammatory phenotype [78,79]. Secondly, it has been suggested that each additional day of antibiotic therapy in the first 2 weeks of life in VLBW infants may be associated with an increased BPD rate and severity [80]. This could be explained by the antibiotic-induced decrease in diversity of lung microbiota which has been linked to BPD development [81]. Probiotics are known to restore intestinal microbiota after antibiotic therapy [82] and to produce a strong suppressive effect on airway inflammation [83]. Lastly, poor nutrition is associated with lung underdevelopment and the occurrence of BPD [84]. In experimental NEC, probiotic supplementation reversed the detrimental effects of combined hyperoxia and suboptimal nutrition on lung vascular endothelial growth factor (VEGF) levels, suggesting that this strategy may help improve lung vasculogenesis [85].

In conclusion, our study could not demonstrate any significant effect of probiotic supplementation on the risk of developing of BPD. Given the remarkable theoretical benefits of probiotics supplementation in ameliorating several aspects of BPD pathogenesis and the limitations of the analysis, our data should be seen as a starting point rather than definitive results. The main merit of our study was to collect, for the first time, the available data on the role of probiotic supplementation in the prevention of BPD, and to revise the possible specific mechanisms of action. Nevertheless, further experimental and clinical data are needed to draw more solid conclusions. Particularly, more studies designed to select the optimal probiotic preparation, dosing, and duration of therapy are still needed [29]. These studies should compare probiotic strains that have been reported to be safe and effective in previous trials [73] and include outcomes, such as BPD, which can be indirectly affected by the changes in immunity and nutritional status induced by probiotic supplementation.

## Figures and Tables

**Table 1:** Characteristics of the included studies.

Study	Participants	Sample Size, GA (Weeks), BW (g)		Intervention	Duration of Intervention	Primary Outcome	BPD Definition
		Probiotics	Control				
Akar 2017 [49]	GA ≤ 32 weeks or BW ≤ 1500 g	*n* = 124	*n* = 125	*Lactobacillus reuteri* vs. no probiotics	From first feed until discharge	Neurodevelopmental outcome	BPD36
		GA: 28.9 (2.1)	GA: 28.6 (2.5)				
		BW: 1138 (257)	BW: 1142 (267)				
Al Hosni 2012 [48]	BW 501–1000 g	*n* = 50	*n* = 51	*Lactobacillus rhamnosus* + *Bifidobacterium infantis* vs. no probiotics	Once daily from the time of initiation of enteral feeds, until discharge or 34 weeks PMA	% infants <10th centile at 34 weeks PMA	BPD36
		GA: 25.7 (1.4)	GA: 25.7 (1.4)				
		BW: 778 (138)	BW: 779 (126)				
Costeloe 2016 [62]	GA < 31 weeks	*n* = 650	*n* = 660	*Bifidobacterium breve* BBG-001 vs. placebo	Commenced within 48 hours of birth, until 36 weeks PMA or discharge	NEC ≥ stage 2, LOS, death	BPD36, Severe BPD
		GA (median): 28.0 (IQR: 26.1–29.4)	GA (median): 28.0 (IQR: 26.1–29.6)				
		BW: 1039 (312)	BW: 1043 (317)				
Demirel 2013 [50]	GA ≤ 32 weeks and BW ≤ 1500 g	*n* = 135	*n* = 136	Saccharomyces *boulardii* vs. no probiotics	Once daily from the time of initiation of enteral feeds, until discharge	NEC ≥ stage 2 or death	BPD28
		GA: 29.4 (2.3)	GA: 29.2 (2.5)				
		BW: 1164 (261)	BW: 1131 (284)				
Dilli 2015 [51]	GA < 32 weeks and BW < 1500 g	*n* = 100	*n* = 100	*Bifidobacterium lactis* vs. placebo	From day 8 of life, once daily until discharge or a maximum of 8 weeks	NEC ≥ stage 2	BPD28, BPD36
		GA: 28.8 (1.9)	GA: 28.2 (2.2)				
		BW: 1236 (212)	BW: 1147 (271)				
Fujii 2006 [52]	GA < 34 weeks	*n* = 11	*n* = 8	*B. breve* M-16V vs. placebo	From several hours after birth until discharge	Serum cytokine levels and expression of Transforming growth factor beta signaling Smad molecules	BPD28
		GA: 31.3 (3.2)	GA: 31.2 (2.0)				
		BW: 1378 (365)	BW: 1496 (245)				
Jacobs 2013 [53]	GA < 32 weeks and BW < 1500 g	*n* = 548	*n* = 551	*B. infantis* + Saccharomyces *thermophilus* + *B. lactis* vs. placebo	From enteral feed ≥ 6 mL/day until discharge or term corrected age.	LOS	BPD28, BPD36
		GA: 27.9 (2.0)	GA: 27.8 (2.0)				
		BW: 1063 (259)	BW: 1048 (260)				
Lin 2008 [54]	GA <34 weeks and BW < 1500 g	*n* = 217	*n* = 217	*Lactobacillus* *acidophilus* + *Bifidobacterium* bifidum vs. no probiotics	From first feeding, for 6 weeks.	Death or NEC ≥ Stage 2	BPD36
		BW: 1029 (246)	BW: 1077 (214)				
Manzoni 2009 [55]	BW < 1500 g	*n* = 151	*n* = 168	*L*. *rhamnosus* GG + lactoferrin vs. placebo	From day 3 of life, for 6 weeks or until discharge	LOS	BPD36
		GA: 29.8 (2.8)	GA: 29.5 (3.2)				
		BW: 1138 (253)	BW: 1109 (269)				
Saengtawesin 2014 [56]	GA ≤ 34 weeks and BW ≤ 1500 g	*n* = 31	*n* = 29	*L. acidophilus* + *B. bifidum* vs. no probiotics	From first enteral feed until 6 weeks of age or discharge	NEC ≥ Stage 2	BPD28
		GA: 31.0 (1.8)	GA: 30.6 (1.8)				
		BW: 1250 (179)	BW: 1208 (199)				
Sari 2012 [57]	GA <33 or BW < 1500 g	*n* = 86	*n* = 88	*Lactobacillus sporogenes* vs. no probiotics	From first enteral feed until discharge	Growth and neurodevelopment at 18–22 months	BPD36
		GA: 29.7 (2.5)	GA: 29.8 (2.3)				
		BW: 1241 (264)	BW: 1278 (273)				
Serce 2013 [58]	GA ≤ 32 weeks and BW ≤ 1500 g	*n* = 104	*n* = 104	*S. boulardii* vs. placebo	From first enteral feed until discharge	NEC ≥ Stage 2 or death or LOS	BPD36
		GA: 28.8 (2.2)	GA: 28.7 (2.1)				
		BW: 1126 (232)	BW: 1162 (216)				
Stratiki 2007 [59]	GA 27–37 weeks	*n* = 41	*n* = 36	*B. lactis* vs. no probiotics	From day 2 to discharge	Intestinal permeability by the sugar absorption test	Undefined
		GA (median): 31 (range: 27–37)	GA (median): 30.5 (range: 26–37)				
		BW (median): 1500 (range: 900–1780)	BW (median): 1500 (range: 700–1900)				
Totsu 2014 [60]	BW < 1500 g	*n* = 153	*n* = 130	*B. bifidum* vs. placebo	Commenced within 48 h of birth and continued until discharge	Postnatal day when enteral feed exceeding 100 mL/kg/day	BPD28, BPD36
		GA: 28.6 (2.9)	GA: 28.5 (3.3)				
		BW: 1016 (289)	BW: 998 (281)				
Underwood 2009 (CUL) [61] ^1^	GA < 35 weeks and BW 750–2000 g	*n* = 30	*n* = 29	*L. rhamnosus* GG + inulin vs. placebo	From first feed until 28 days or discharge	Weight gain	BPD36
		GA: 29.5 (2.6)	GA: 29.3 (2.6)				
		BW: 1394 (356)	BW: 1393 (363)				
Underwood 2009 (PBP) [61] ^1^	GA < 35 weeks and BW 750–2000 g	*n* = 31	*n* = 29	*L. acidophilus* + *Bifidobacterium longum* + *B. bifidum* + *B. infantis* + inulin vs. placebo	From first feed until 28 days or discharge	Weight gain	BPD36
		GA: 30.2 (2.4)	GA: 29.3 (2.6)				
		BW: 1461 (372)	BW: 1393 (363)				

^1^ Culturelle^®^ (CUL) and ProBioPlus DDS^®^ (PBP) were the names assigned by the authors to the probiotic preparations. BPD: bronchopulmonary dysplasia; BPD28: bronchopulmonary dysplasia, defined as oxygen dependence at 28 days of life; BPD36: bronchopulmonary dysplasia, defined as oxygen dependence at 36 weeks post-menstrual age; Severe BPD: defined as any baby at 36 weeks PMA still receiving mechanical ventilator support or in at least 30% oxygen or more than 0.1 L/min of low flow oxygen. BW: birth weight; GA: gestational age; IQR: interquartile range; NEC: necrotizing enterocolitis; PMA: postmenstrual age; LOS: late-onset sepsis. Data for GA and BW given in mean (standard deviation), unless noted otherwise.

**Table 2:** Subgroup analysis of probiotics and risk of BPD.

Subgroup	*k*	BPD Definition	Sample Size	MH RR	95% CI	*p*
Studies where *Lactobacillus* was part of the supplementation	6	BPD36	1335	1.01	0.80–1.29	0.904
Studies where *Bifidobacterium* was part of the supplementation	5	BPD28	1601	1.00	0.90–1.11	0.999
	4	BPD36	2781	1.10	0.90–1.33	0.346
Single-strain supplementation	4	BPD28	773	0.97	0.79–1.18	0.763
	7	BPD36	2372	1.08	0.88–1.32	0.480
Multiple-strain supplementation	2	BPD28	1159	1.01	0.90–1.13	0.829
	5	BPD36	2012	1.06	0.87–1.29	0.574
Studies with infants mean BW < 250 g	5	BPD28	1913	1.01	0.91–1.11	0.893
	9	BPD36	4091	1.08	0.96–1.22	0.195
Studies with low risk of bias on random sequence generation and allocation concealment	9	BPD36	3752	1.08	0.97–1.19	0.155
Studies with low risk of bias on incomplete outcome data	10	BPD36	3927	1.09	0.96–1.23	0.188
Studies with low risk of bias on selective reporting	9	BPD36	3493	1.06	0.94–1.18	0.344

BPD: bronchopulmonary dysplasia; BPD28: bronchopulmonary dysplasia, defined as oxygen dependence at 28 days of life; BPD36: bronchopulmonary dysplasia, defined as oxygen dependence at 36 weeks post-menstrual age; CI: confidence interval; *k*: number of studies included; MH RR: Mantel–Haenszel risk ratio.

**Table 3:** NEC, LOS and mortality in the included studies.

Study	NEC (Affected/Total)		NEC Definition	LOS (Affected/Total)		LOS Definition	Mortality (Affected/Total)		Mortality Definition
	Probiotics	Control		Probiotics	Control		Probiotics	Control	
Akar 2017 [49]	1/124	6/125	NEC stage ≥ 2	8/124	19/125	Culture-proven sepsis	14/200	16/200	Death before 18–24 month follow-up
Al Hosni 2012 [48]	2/50	2/51	NEC stage ≥ 2	13/50	16/51	Culture-proven sepsis	3/50	4/51	Death before 34 weeks PMA
Costeloe 2016 [62]	61/650	66/660	NEC stage ≥ 2	73/650	77/660	Culture-proven sepsis > 72 h	54/650	56/660	Death during primary hospitalization
Demirel 2013 [50]	6/135	7/136	NEC stage ≥ 2	20/135	21/136	Culture-proven sepsis	5/135	5/136	Death after 7 days of life
Dilli 2015 [51]	2/100	18/100	NEC stage ≥ 2	8/100	13/100	Culture-proven sepsis > 72 h	3/100	12/100	Not defined
Fujii 2006 [52]	0/11	0/8	Not defined	1/11	1/8	Not defined	0/11	0/8	Death during primary hospitalization
Jacobs 2013 [53]	11/548	24/551	NEC stage ≥ 2	72/548	89/551	Culture-proven sepsis > 48 h	30/548	31/551	Death during primary hospitalization
Lin 2008 [54]	4/217	14/217	NEC stage ≥ 2	40/217	24/217	Culture-proven > 72h	2/217	9/217	Death during intervention (6 weeks)
Manzoni 2009 [55]	0/151	10/168	NEC stage ≥ 2	7/151	29/168	Culture-proven sepsis > 72 h	6/153	12/168	Death during primary hospitalization
Saengtawesin 2014 [56]	1/31	1/29	NEC stage ≥ 2	2/31	1/29	Not defined	0/31	0/29	Death during primary hospitalization
Sari 2012 [57]	3/86	7/88	NEC stage ≥ 2	24/86	19/88	Not defined	5/110	8/111	Death before 18 to 22 months of age
Serce 2013 [58]	7/104	7/104	NEC stage ≥ 2	19/104	25/104	Culture-proven sepsis	5/104	4/104	Death during primary hospitalization
Stratiki 2007 [59]	0/41	3/36	NEC stage ≥ 2	0/41	3/36	Culture-proven sepsis	0/41	0/36	Not defined
Totsu 2014 [60]	0/153	0/130	NEC stage ≥ 2	6/153	10/130	Culture-proven sepsis ≥ 1 week	2/153	0/130	Death during primary hospitalization
Underwood 2009 (CUL) [61] ^1^	1/30	1/29	NEC stage ≥ 2	4/30	4/29	Culture-proven sepsis > 72 h	0/30	0/29	Death during primary hospitalization
Underwood 2009 (PBP) [61] ^1^	1/31	1/29	NEC stage ≥ 2	2/31	4/29	Culture-proven sepsis > 72 h	0/31	0/29	Death during primary hospitalization

^1^ Culturelle (CUL) and ProBioPlus DDS (PBP) were the names assigned by the authors to the probiotic preparations. LOS: late-onset sepsis; NEC: necrotizing enterocolitis.

**Table 4:** Random effects meta-analysis of probiotics and LOS, NEC and mortality.

Meta-Analysis	*k*	BPD Definition	MH Risk Ratio	95% CI	Z	*p*	Heterogeneity		
							Q	*p*	*I*^2^
Probiotics NEC	15	All	0.52	0.33 to 0.81	−2.88	0.004	22.0	0.055	40.9%
	7	BPD28	0.40	0.18 to 0.88	−2.29	0.022	6.3	0.175	37.0%
	12	BPD36	0.48	0.29 to 0.81	−2.73	0.006	20.4	0.025	51.1%
Probiotics LOS	15	All	0.82	0.65 to 1.03	−1.73	0.084	26.8	0.031	44.0%
	7	BPD28	0.79	0.63 to 1.00	−1.93	0.054	3.6	0.72	0.0%
	12	BPD36	0.80	0.62 to 1.04	−1.70	0.090	24.5	0.011	55.1%
Probiotics mortality	11	All	0.84	0.66 to 1.07	−1.38	0.169	10.4	0.410	3.4%
	4	BPD28	0.78	0.37 to 1.66	−0.65	0.518	5.2	0.155	42.8%
	10	BPD36	0.82	0.62 to 1.07	−1.45	0.146	10.3	0.328	12.5%

BPD: bronchopulmonary dysplasia; BPD28: bronchopulmonary dysplasia, defined as oxygen dependence at 28 days of life; BPD36: bronchopulmonary dysplasia, defined as oxygen dependence at 36 weeks post-menstrual age; CI: confidence interval; *k*: number of studies included; LOS: late onset-sepsis; MH: Mantel–Haenszel; NEC: necrotizing enterocolitis.

## Supplementary Materials

Supplementary File 1

## Appendix A

**Table A1:** Risk of bias assessment of studies included in meta-analysis.

Study	Random Sequence Generation	Allocation Concealment	Blinding of Participants and Personnel	Blinding of Outcome Assessment	Incomplete Outcome Data	Selective Reporting	Other Bias
Akar 2017 [49]	LR	UR	LR	LR	UR	UR	UR
Al Hosni 2011 [48]	UR	UR	LR	LR	LR	LR	LR
Costeloe 2016 [62]	LR	LR	LR	LR	LR	LR	LR
Demirel 2013 [50]	LR	LR	LR	LR	LR	LR	LR
Dilli 2015 [51]	LR	LR	LR	LR	LR	LR	LR
Fujii 2006 [52]	UR	UR	HR	UR	LR	UR	UR
Jacobs 2013 [53]	LR	LR	LR	LR	LR	LR	LR
Lin 2008 [54]	LR	LR	LR	LR	LR	UR	LR
Manzoni 2009 [55]	LR	UR	LR	LR	LR	LR	LR
Saengtawesin 2014 [56]	UR	UR	HR	HR	UR	UR	UR
Sari 2012 [57]	LR	LR	LR	LR	LR	LR	LR
Serce 2013 [58]	LR	LR	LR	LR	UR	UR	LR
Stratiki 2007 [59]	UR	UR	LR	LR	UR	UR	LR
Totsu 2014 [60]	LR	UR	LR	LR	LR	LR	LR
Underwood 2009 [61]	LR	LR	LR	LR	LR	LR	LR

HR: High risk of bias; LR: Low risk of bias; UR: Unclear risk of bias.

**Table A2:** Sensitivity analyses for BPD28: results of random effects meta-analyses when removing one study.

Removed Study	Statistics with Study Removed				
	MH RR	Lower Limit 95% CI	Upper Limit 95% CI	*Z*-Score	*p*
Demirel 2013 [50]	1.01	0.92	1.12	0.21	0.832
Dilli 2015 [51]	1.02	0.92	1.13	0.37	0.708
Fujii 2006 [52]	1.01	0.91	1.11	0.15	0.878
Jacobs 2013 [53]	1.04	0.86	1.25	0.38	0.703
Saengtawesin 2014 [56]	1.00	0.90	1.10	−0.09	0.931
Totsu 2014 [60]	0.99	0.89	1.10	−0.22	0.829
Stratiki 2007 [59]	1.01	0.91	1.11	0.11	0.916

BPD28: bronchopulmonary dysplasia, defined as oxygen dependence at 28 days of life; CI: confidence interval; MH RR: Mantel–Haenszel risk ratio.

**Table A3:** Sensitivity analyses for BPD36: results of random effects meta-analyses when removing one study.

Removed Study	Statistics with Study Removed				
	MH RR	Lower Limit 95% CI	Upper Limit 95% CI	*Z*-Score	*p*
Akar 2017 [49]	1.09	0.97	1.21	1.45	0.146
Al Hosni 2012 [48]	1.09	0.97	1.23	1.48	0.138
Costeloe 2016 [62]	1.07	0.93	1.22	0.94	0.350
Dilli 2015 [51]	1.08	0.97	1.21	1.43	0.153
Jacobs 2013 [53]	1.09	0.96	1.24	1.32	0.188
Lin 2008 [54]	1.04	0.93	1.17	0.69	0.488
Manzoni 2009 [55]	1.08	0.96	1.20	1.32	0.187
Sari 2012 [57]	1.07	0.96	1.20	1.25	0.213
Serce 2013 [58]	1.07	0.96	1.20	1.26	0.209
Totsu 2014 [60]	1.05	0.93	1.17	0.76	0.448
Underwood 2009 (CUL) [61] ^1^	1.08	0.97	1.20	1.34	0.179
Underwood 2009 (PBP) [61] ^1^	1.08	0.97	1.20	1.36	0.173

^1^ Culturelle (CUL) and ProBioPlus DDS (PBP) were the names assigned by the authors to the probiotic preparations. BPD36: bronchopulmonary dysplasia, defined as oxygen dependence at 36 weeks post-menstrual age; CI: confidence interval; MH RR: Mantel–Haenszel risk ratio.
